# Invasive Pneumococcal Disease and 7-Valent Pneumococcal Conjugate Vaccine, the Netherlands

**DOI:** 10.3201/eid1811.120329

**Published:** 2012-11

**Authors:** Anna M.M. van Deursen, Suzan P. van Mens, Elisabeth A.M. Sanders, Bart J.M. Vlaminckx, Hester E. de Melker, Leo M. Schouls, Sabine C. de Greeff, Arie van der Ende, on behalf of the Invasive Pneumococcal Disease Sentinel Surveillance Laboratory Group

**Keywords:** invasive pneumococcal disease, *Streptococcus pneumoniae*, epidemiology, 7-valent pneumococcal conjugate vaccine, PCV7, age-specific rates, surveillance, herd immunity, the Netherlands, bacteria, vaccines

## Abstract

Disease incidence and case fatality rates declined 4 years after introduction of the vaccine.

## Medscape CME Activity

Medscape, LLC is pleased to provide online continuing medical education (CME) for this journal article, allowing clinicians the opportunity to earn CME credit. 

This activity has been planned and implemented in accordance with the Essential Areas and policies of the Accreditation Council for Continuing Medical Education through the joint sponsorship of Medscape, LLC and Emerging Infectious Diseases. Medscape, LLC is accredited by the ACCME to provide continuing medical education for physicians.

Medscape, LLC designates this Journal-based CME activity for a maximum of 1 *AMA PRA Category 1 Credit(s)*^TM^. Physicians should claim only the credit commensurate with the extent of their participation in the activity.

All other clinicians completing this activity will be issued a certificate of participation. To participate in this journal CME activity: (1) review the learning objectives and author disclosures; (2) study the education content; (3) take the post-test with a 70% minimum passing score and complete the evaluation at www.medscape.org/journal/eid; (4) view/print certificate.

**Release date: October 19, 2012; Expiration date: October 19, 2013**

## Learning Objectives

Upon completion of this activity, participants will be able to:

- Analyze previous research into the effects of 7-valent pneumococcal conjugate vaccine (PCV7)
- Compare the effects of PCV7 on different continents
- Distinguish age groups most affected by PCV7
- Evaluate the clinical presentation and outcomes of IPD after introduction of PCV7.

## CME Editor

**Claudia Chesley, **Technical Writer/Editor, *Emerging Infectious Diseases. Disclosure: Claudia Chesley has disclosed no relevant financial relationships.*

## CME Author

**Charles P. Vega, MD, **Health Sciences Clinical Professor; Residency Director, Department of Family Medicine, University of California, Irvine. *Disclosure: Charles P. Vega, MD, has disclosed no relevant financial relationships.*

## Authors

*Disclosures:* ***Anna M.M. van Deursen*** *;* ***Suzan P. van Mens, MD*** *;* ***Bart J.M. Vlaminckx, MD*** *,* ***PhD*** *;* ***Hester E. de Melker*** *;* ***Leo M. Schouls*** *; and* ***Sabine C. de Greeff, MSc*** *, have disclosed no relevant financial relationships.* ***Elisabeth A.M. Sanders, MD, PhD*** *, has disclosed the following relevant financial relationships: served as an advisor or consultant for Pfizer, GSK; received grants for clinical research from Pfizer, GSK.* ***Arie van der Ende, PhD*** *, has disclosed the following relevant financial relationships: served as an advisor or consultant for Pfizer, GSK; received grants for clinical research from Pfizer, GSK.*
