# Supplementary material for: Invasive Pneumococcal Disease and 7-Valent Pneumococcal Conjugate Vaccine, the Netherlands
Source: Emerg Infect Dis. 2012 Nov;18(11):1729–37. doi: 10.3201/eid1811.120329 (PMC3559145; doi:10.3201/eid1811.120329)
Supplement: Technical Appendix — Clinical characteristics of patients with invasive pneumococcal disease before and after introduction of 7-valent pneumococcal conjugate vaccine, nationwide collection of pneumococcal isolates, and incidence rate ratio of serotype-specific invasive pneumococcal disease among patients >65 years of age, the Netherlands. [file 12-0329-Techapp-s1.pdf]

Article DOI: http://dx.doi.org/10.3201/eid1811.120329

Invasive Pneumococcal Disease and 7-Valent Pneumococcal Conjugate Vaccine, the Netherlands

Technical Appendix

Technical Appendix Table. Clinical characteristics for persons with invasive pneumococcal disease, by age group, before and after introduction of PCV7 vaccine, the Netherlands, June 2004–May 2010*

	All serotypes			PCV7			Non-PCV7		
Age group, y, characteristic	Pre	Post	p value	Pre	Post	p value	Pre	Post	p value
<5									
Cases total, n	93	38		65	3		28	35	
Comorbidities									
Immunocompromising condition†, n (%)	4 (4)	3 (8)	NS	2 (3)	0 (0)	NS	2 (7)	3 (9)	NS
Any comorbidity‡, n (%)	31 (33)	7 (18)	NS	22 (34)	0 (0)	NS	9 (32)	7 (20)	NS
Disease course/outcome									
ICU admission, n (%)	15 (16)	5 (13)	NS	11 (17)	0 (0)	NS	4 (14)	5 (14)	NS
Length of stay, median (IQR)	8.0 (5.0–12.0)	8.0 (4.0–12.5)	NS	8.0 (4.5–12.5)	10.0 (3.0–∞)	NS	8.0 (4.8–12.0)	7.5 (4.0–11.8)	NS
Case-fatality, n (%)	5 (5)	2 (5)	NS	4 (6)	0 (0)	NS	1 (4)	2 (6)	NS
Mortality rate, cases/100,000	1.0	0.4	NS	0.8	0.0	NS	0.2	0.4	NS
5–64									
Cases total, n	457	481		181	108		276	373	
Comorbidities									
Immunocompromising condition†, n (%)	88 (19)	102 (21)	NS	27 (15)	30 (28)	0.008§	61 (22)	72 (19)	NS
Any comorbidity‡, n (%)	257 (56)	272 (57)	NS	106 (59)	63 (58)	NS	151 (55)	209 (56)	NS
Disease course /outcome									
ICU admission, n (%)	106 (23)	120 (25)	NS	46 (25)	27 (25)	NS	60 (22)	93 (25)	NS
Length of stay, median (IQR)	10.0 (6.0–17.0)	8.0 (5.0–15.0)	<0.001§	10.0 (6.0–17.0)	8.0 (5.0–14.0)	0.027§	10.0 (6.0–17.0)	8.0 (5.0–15.0)	0.006§
Case-fatality, n (%)	41 (9)	31 (6)	NS	18 (10)	9 (8)	NS	23 (8)	22 (6)	NS
Mortality rate, cases/100,000	0.6	0.5	NS	0.3	0.1	NS	0.4	0.3	NS
≥65									
Cases total, n	666	625		319	157		346	468	
Comorbidities									
Immunocompromising condition†, n (%)	124 (19)	150 (24)	0.018§	59 (18)	43 (27)	0.026§	65 (19)	107 (23)	NS
Any comorbidity‡, n (%)	529 (79)	509 (81)	NS	248 (78)	127 (81)	NS	281 (81)	382 (82)	NS
Disease course /Outcome									
ICU admission, n (%)	137 (21)	118 (19)	NS	58 (18)	33 (21)	NS	79 (23)	85 (18)	NS

Length of hospital stay, median (IQR)	13.0 (8.0–20.0)	10.0 (6.0–18.0)	<0.001§	13.0 (8.0–20.0)	10.0 (6.0–17.0)	0.001§	13.0 (8.0–21.0)	11.0 (6.0–18.0)	0.005§
Case-fatality, n (%)	148 (22)	102 (16)	0.007§	70 (22)	35 (22)	NS	78 (23)	67 (14)	0.003§
Mortality rate, cases/100,000	12.8	8.1	<0.001§	6.1	2.8	<0.001§	6.8	5.4	NS

*Cases = number of cases included in the surveillance study (covering ≈25% of the Dutch population); Pre = pre-implementations period (June 2004-May 2006); Post = late post-implementation period (June 2008-May 2010). PCV7, 7-valent pneumococcal conjugate vaccine; ICU, intensive care unit; IQR, interquartile range; NS, not significant.

†Immunocompromising condition: primary immunodeficiency, HIV/AIDS, lymphoma, leukemia, myeloma, solid organ or stem cell transplantation, current immunosuppressive therapy for malignancy or autoimmune disease, asplenia/splenectomy, sickle cell disease and renal insufficiency (need for dialysis and nephrotic syndrome).

‡Any comorbidity: malignancies (within the previous 5 y) not considered to be immunocompromising, chronic pulmonary disease (chronic obstructive pulmonary disease and asthma), diabetes mellitus, cardiovascular disease (myocardial infarction, coronary artery condition, stroke/transient ischemic attack (TIA), cardiomyopathy, heart failure, heart valve disease, and/or presence of cerebral/abdominal/thoracic aneurysms), thyroid disease, liver disease, intravenous drug use, long-term alcohol abuse, cerebrospinal fluid leak, recent physical trauma/skull fracture and for children premature birth (<37 weeks for children 0–1 y old and <32 for children 0–4 y old).

§Significant difference ($p < 0.05$) between pre- and post-implementation period calculated by $\chi 2$ test (% of cases), Mann-Whitney U (median length hospital of stay) or incidence rate ratio (mortality rate).

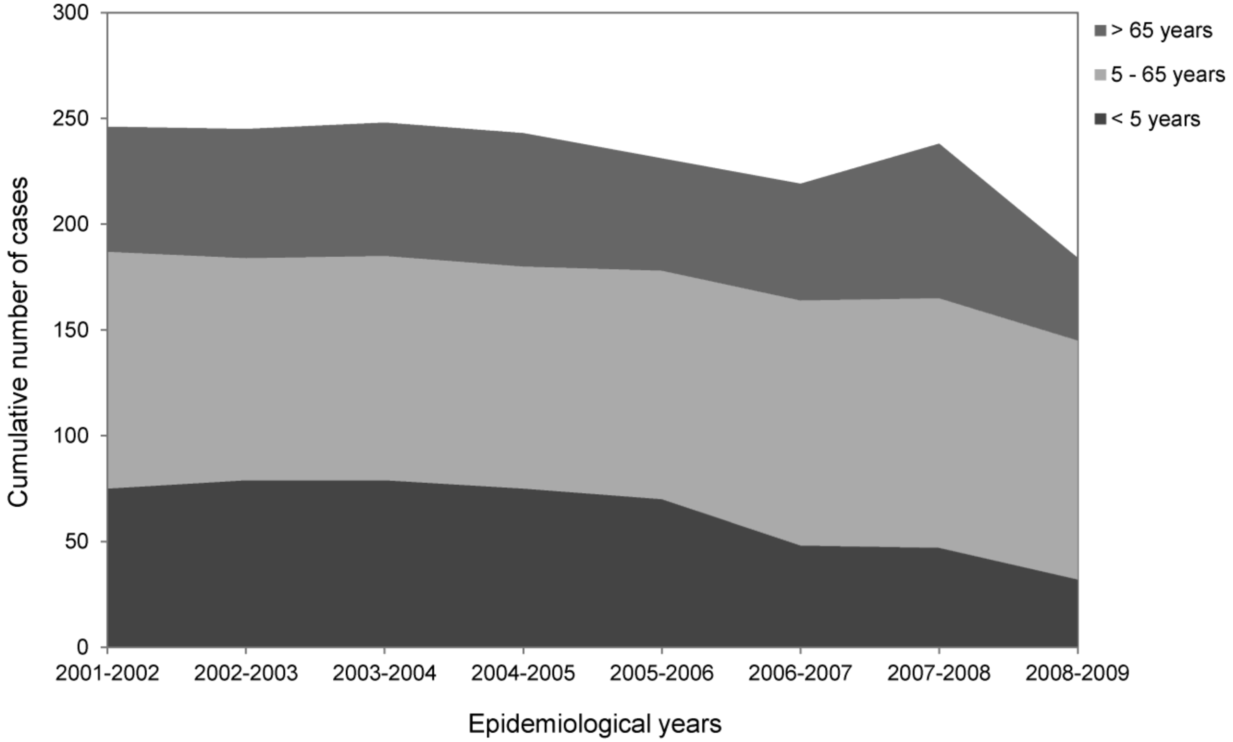

Technical Appendix Figure 1. Nationwide collection of pneumococcal isolates (from cerebrospinal fluid), the Netherlands, 2001–02 through 2008–09. Epidemiologic years, June 1–May 31 of the succeeding year.

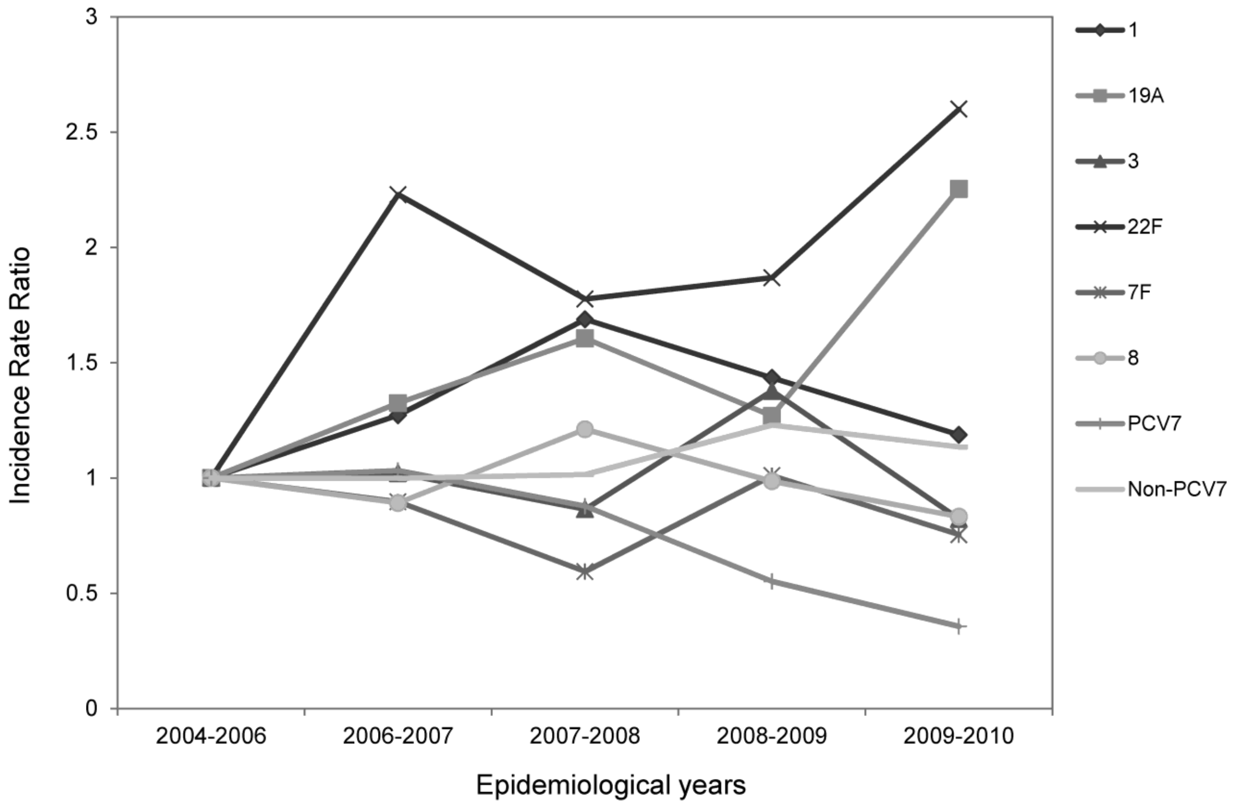

Technical Appendix Figure 2. Incidence rate ratio of serotype-specific invasive pneumococcal disease among patients ≥65 years of age, the Netherlands, 2004-2010. Epidemiologic years, June 1–May 31 of the succeeding year. Incidence rate ratios (IRRs) calculated by using 2004-2006 as reference period (IRR=1.00).
