# Supplementary material for: Invasive Pneumococcal Disease and 7-Valent Pneumococcal Conjugate Vaccine, the Netherlands
Source: Emerg Infect Dis. 2012 Nov;18(11):1729–37. doi: 10.3201/eid1811.120329 (PMC3559145; doi:10.3201/eid1811.120329)
Supplement: Related texts

# Research article: Invasive Pneumococcal Disease and 7-Valent Pneumococcal Conjugate Vaccine, the Netherlands

**Authors:** Anna M.M. van Deursen, Suzan P. van Mens, Elisabeth A.M. Sanders, Bart J.M. Vlaminckx, Hester E. de Melker, Leo M. Schouls, Sabine C. de Greeff, Arie van der Ende, on behalf of the Invasive Pneumococcal Disease Sentinel Surveillance Laboratory Group

## Abstract

In the Netherlands, the national immunization program includes 7-valent pneumococcal conjugate vaccine (PCV7) for all newborns born after April 1, 2006. We compared the incidence of invasive pneumococcal disease (IPD) and patient and disease characteristics before PCV7 introduction (June 2004–June 2006) with those after PCV7 introduction (June 2008–June 2010). Culture-confirmed IPD cases were identified by 9 sentinel laboratories covering ≈25% of the Dutch population. Significant declines in overall IPD incidence were observed in children <2 (60%) and in persons >65 (13%) years of age. A trend toward gradual increases in non–PCV7 serotype IPD infections was observed in all age groups; the largest increases were among persons 50–64 (37%) and >65 (25%) years of age. In adults, the proportion of immunocompromised persons increased among IPD patients. Overall, deaths from IPD decreased from 16% to 12% because of a lower case-fatality rate for persons with non–PCV7 serotype IPD.

*Streptococcus pneumoniae* is a major cause of severe invasive infections, such as meningitis, invasive pneumonia, and other bloodstream infections. The highest incidence rates for such infections are for infants and elderly persons (*1*).

Since 2001, many high-income countries included the 7-valent pneumococcal conjugate vaccine (PCV7; Prevenar; Pfizer Pharmaceuticals, Pearl River, NY, USA) in their national immunization programs for newborns (*2*). In general, within a few years after the introduction of PCV7, the age group targeted for vaccination and unvaccinated adults showed a dramatic decrease in invasive pneumococcal disease (IPD) caused by the 7 vaccine serotypes (*2*–*5*). However, at the same time, the incidence of non-PCV7 serotype IPD increased (*3*,*4*,*6*,*7*).

The overall benefit of PCV7 varies by country, perhaps as a result of differences in surveillance methods and the maturity of vaccination programs (*8*). For all age groups, the overall reduction in IPD incidence is greater in the United States than in European countries; the great reduction in the United States is a result of a decrease in PCV7-serotype IPD in adults and less replacement of PCV7-serotype by non–PCV7 serotype IPD in children and older adults (*3*,*4*,*7*). The United States began using PCV7 in 2000, but many European countries did not begin using the vaccine until after 2005–2006, and they have experienced less protection from indirect herd protection (herd immunity). Furthermore, not all European countries implemented a catch-up program for children <5 years of age; catch-up programs speed up eradication of vaccine serotypes. Geographic variations in circulation of PCV7 serotypes before the implementation of routine vaccination also caused differences in the relative proportion of IPD covered by the vaccine (*7*,*8*).

In addition, the benefits of vaccination with PCV7 may have been biased, for example, by changes in the directive for blood culture after 2000, as in the United States (*9*,*10*), and by enhanced surveillance, as reported for England and Wales (*4*). Unlike studies in the United States, studies in Europe, particularly Dutch surveillance studies, have focused almost exclusively on patients requiring hospitalization for severe IPD and who often had other underlying illnesses (*11*,*12*). This difference in reporting leads to different baseline incidence rates and may affect the observed net benefit of vaccination (*13*). For example, compared with healthy persons of the same age, US adults with comorbid conditions benefited less from the indirect effects of PCV7 because of an increase in non–PCV7 serotype IPD after introduction of the vaccine (*14*). Differences in the directive for blood culture and patient populations under surveillance can partly explain the differences in results from use of PCV7.

The invasive disease potential of *S. pneumoniae* and the population at risk for IPD differs by serotype (*12*,*13*,*15*). Therefore, shifts in circulating serotypes may change the clinical manifestations of IPD, the population segment most at risk for infection, and the disease course and outcome. We investigated these issues and changes in IPD incidence in the Netherlands 4 years after a PCV7 vaccine program was implemented and compared our findings with those from the years just before introduction of the vaccine.

## Methods

### Pneumococcal Vaccination in the Netherlands

PCV7 was introduced into the Dutch national immunization program in June 2006 and was recommended for children born after April 1, 2006, at 2, 3, 4, and 11 months of age (*16*). Vaccination uptake is 94%–95% among Dutch infants (*17*). Use of the 23-valent pneumococcal polysaccharide vaccine is restricted to persons at high-risk for IPD (e.g., persons with asplenia or Hodgkin lymphoma); uptake in elderly persons is negligible (<1%) (*18*).

### Surveillance Data

For this study, we registered all persons with a diagnosis of culture-confirmed IPD during June 1, 2008–May 31, 2010 (late post-implementation period) and all case-patients from previous Dutch IPD surveillance studies during June 1, 2004–May 31, 2006 (pre-implementation period) (*1*) and June 1, 2006–May 31, 2008 (post-implementation period) (*11*). All study procedures were the same as those used in the previous studies (*11*).

Nine sentinel laboratories identified IPD case-patients, which were defined as patients for whom *S. pneumoniae* was isolated from blood or cerebrospinal fluid (CSF) samples. The laboratories submitted all invasive pneumococcal isolates to the Netherlands Reference Laboratory for Bacterial Meningitis (NRLBM, Academic Medical Center, Amsterdam, the Netherlands) for typing and characterization. We selected the laboratories on the basis of their geographic distribution throughout the country and their reliability for submitting isolates (*1*,*11*). The laboratories were estimated to cover a representative cohort of ≈25% of the Dutch population (≈4.1 million inhabitants, including ≈0.6 million adults >65 years of age). In addition, ≈25% of the other meningitis-causing bacterial isolates that were submitted to NRLBM during the study period were submitted by the 9 sentinel laboratories.

At the NRLBM, co-agglutination was used to type the pneumococcal isolates and the capsular swelling method (Quellung reaction), using antisera (Statens Serum Institute, Copenhagen, Denmark), including serotype 6C, was used for serotyping. For isolates collected before June 2008, serotype 6C was determined by using PCR and antisera. The serotypes were grouped in either PCV7 serotypes (4, 6B, 9V, 14, 18C, 19F, 23F) or non–PCV7 serotypes (all other serotypes, including 6A).

### Clinical Characteristics

Trained medical students, using a standardized data collection form, retrospectively extracted the following information for all case-patients from hospital records, as described (*1*,*11*): patient characteristics, clinical syndrome, comorbidity, and disease course and outcome. We subdivided comorbid conditions as immunocompromising or nonimmunocompromising and categorized clinical syndromes as meningitis, invasive pneumonia, bacteremia with other focus, and bacteremia without focus, as described (*1*). Information on disease course and outcome included the length of hospital stay, admission to an intensive care unit, and death (i.e., in-hospital death and/or death within 30 days after first reported blood/CSF culture positive for *S. pneumoniae*). Cases without clinical information were excluded from all analyses.

### Statistical Analyses

National population coverage was ≈25% by the sentinel laboratories; thus, we estimated annual IPD incidence rates per 100,000 inhabitants by dividing the total number of IPD cases in a specific epidemiologic year by 25% of the total Dutch population. Epidemiologic years were defined as June 1st–May 31 of the succeeding year. We used the population on January 1 of each consecutive year as the population at risk for infection (StatLine, www.cbs.nl/en-GB/menu/cijfers/statline/zelf-tabellen-maken/default.htm), assuming a stable population throughout the year.

We assessed the effect of vaccination by determining the incidence rate ratio. The assessment was done by comparing incidences in the late post-implementation period (2008–2010) with those in the pre-implementation period (2004–2006); we also determined 95% CIs.

To evaluate any changes in population at risk, we compared the proportion of patients with comorbid conditions in the pre- and late post-implementation periods. We also determined changes in disease course (intensive care unit admission, median length of hospital stay, and death). Differences in percentages were compared by using the χ^2^ test, and differences in median length of hospital stay were compared by using the Mann-Whitney U test.

All analyses were stratified by age group (<2, 2–4, 5–17, 18–50, 50–64, and >65 years) and by serotype group (PCV7/non–PCV7). All p values <0.05 were considered statistically significant.

## Results

### Overview

In the late post-implementation period (June 1, 2008–May 31, 2010), a total of 1,196 pneumococcal isolates from CSF and blood samples were submitted to the NRLBM by the 9 sentinel laboratories; this number compares with 1,297 and 1,352 isolates submitted during the pre- and early post-implementation periods, respectively. In the late post-implementation period, clinical characteristics were available for 1,144 (96%) case-patients, compared with 1,216 (94%) in the pre-implementation period and 1,304 (96%) in the early post-implementation period (Table 1).

**Table 1:** Incidence of invasive pneumococcal disease before and after implementation of a PCV7 vaccination program, the Netherlands, June 2004–May 2010*

Age group, y	Vaccination period†									Late post- vs. pre-implementation period		
	Pre-implementation			Early post- implementation			Late post-implementation					
	No. cases	Incidence		No. cases	Incidence		No. cases	Incidence		IRR	95% CI	p value
All serotypes												
All ages	1,216	14.9		1,304	15.9		1,144	13.8		0.93	0.86–1.01	NS
<2	68	35.0		42	22.8		26	14.1		0.40	0.26–0.63	**<0.001**
2–4	25	8.2		26	8.9		12	4.3		0.52	0.26–1.04	NS
5–17	23	1.8		22	1.7		23	1.8		1.00	0.56–1.78	NS
18–49	181	4.9		209	5.8		197	5.5		1.11	0.91–1.36	NS
50–64	253	16.4		292	18.3		261	15.8		0.96	0.81–1.14	NS
>65	666	57.7		713	59.6		625	49.9		0.87	0.78–0.97	**0.009**
PCV7 serotypes												
All ages	565	6.9		561	6.9		268	3.2		0.47	0.40–0.54	**<0.001**
<2	48	24.7		15	8.1		0	0.0		0	NA	**<0.001**
2–4	17	5.6		17	5.8		3	1.1		0.19	0.06–0.66	**0.003**
5–17	11	0.9		4	0.3		4	0.3		0.36	0.12–1.14	NS
18–49	56	1.5		66	1.8		48	1.3		0.87	0.59–1.29	NS
50–64	114	7.4		129	8.1		56	3.4		0.46	0.33–0.63	**<0.001**
>65	319	27.6		330	27.6		157	12.5		0.45	0.37–0.55	**<0.001**
Non–PCV7 serotypes												
All ages	650	8.0		741	9.1		876	10.6		1.33	1.20–1.47	**<0.001**
<2	20	10.3		27	14.7		26	14.1		1.37	0.77–2.46	NS
2–4	8	2.6		9	3.1		9	3.2		1.22	0.47–3.18	NS
5–17	12	0.9		18	1.4		19	1.5		1.58	0.77–3.26	NS
18–49	125	3.4		142	3.9		149	4.1		1.22	0.96–1.54	NS
50–64	139	9.0		163	10.2		205	12.4		1.37	1.11–1.70	**0.004**
>65	346	30.0		382	32.0		468	37.4		1.25	1.09–1.43	**0.002**

*Cases are number of patients included in a study covering ≈25% of the Dutch population; incidence is number of cases/100,000 persons. Three pneumococcal isolates (1 in the pre- and 2 in the early post-implementation period) were either not typeable or typed as a rough strain and, therefore, could not be classified as 7-valent pneumococcal conjugate vaccine (PCV7) or non–PCV7 serotypes. IRR, incidence rate ratio; NS, not significant (p>0.05); NA, not applicable; **boldface**, significant difference (p<0.05). †Vaccination periods: pre-implementation period, June 2004–May 2006; early post-implementation, June 2006–May 2008; late post-implementation period, June 2008–May 2010.

### IPD Incidence and Serotype Distribution

The overall incidence of IPD declined from 14.9 to 13.8 cases/100,000 persons during the pre- and late post-implementation periods, respectively (Table 1). A 60% decline in overall IPD incidence (from 35.0 to 14.1 cases/100,000 persons) was observed in children <2 years of age (i.e., children age-eligible for PCV7 vaccination). A similar but nonsignificant decline was seen in children 2–4 years of age. In the age group with the highest incidence rate, i.e., persons >65 years of age, the overall IPD incidence had a significant decline of 13% (from 57.7 to 49.9 cases/100,000 persons). IPD incidence rates remained unchanged in persons 5–64 years of age.

The overall decline of IPD incidences seen among persons <2 and >65 years of age from the pre- to the late post-implementation period resulted from declines in the incidence of PCV7-serotype IPD of 100% and 55%, respectively (Table 1); in children <2 years of age, no PCV7-serotype IPD cases were reported after June 1, 2008. Of 3 children (2–4 years of age) with PCV7-serotype IPD after June 1, 2008, 2 were born before April 1, 2006 and had not received PCV7. The third patient (2 years of age) experienced a vaccine failure; PCV7–serotype 19F IPD developed even though the child was fully vaccinated with 4 doses of PCV7. The child was previously healthy, without any comorbidity. Overall, infections with all PCV7 serotypes declined significantly, except for infection with serotype 18C, which was already low (Figure).

**Figure:** Serotype distribution of invasive pneumococcal disease in the Netherlands before and after (early and late) introduction of the 7-valent pneumococcal conjugate vaccine (PCV7). The 7 vaccine serotypes and the most prevalent nonvaccine serotypes are shown. The cases represent case-patients included in the study (covering ≈25% of the Dutch population). Gray, pre-implementation period (June 2004–May 2006); white, early post-implementation period (June 2006–May 2008); black, late post-implementation period (June 2008–May 2010); *Significant difference (p<0.05) between pre- and post-implementation periods, calculated by the incidence rate ratio.

However, from the pre- to the late post-implementation period, the overall incidence of non–PCV7 serotype IPD increased by 33% (from 8.0 to 10.6 cases/100,000 persons) (Table 1). IPD incidence due to non–PCV7 serotypes showed an increasing trend in all age groups, and the increase was significant in patients 50–64 and >65 years of age. Non–PCV7 serotypes 1, 19A, 22F, and 23B increased significantly (Figure), although absolute numbers remained relatively small.

### Clinical Characteristics

During all 3 study periods, surveillance data were primarily (97%–98%) for hospitalized IPD patients; the few exceptions were data for patients who visited a hospital emergency department and went home the same day. The distribution of clinical IPD manifestations among patients in different age groups did not change between the pre- and late post-implementation period (Table 2). In children <5 years of age, there was no decline in the incidence of meningitis because of an increase in non–PCV7 serotype meningitis in the late post-implementation period. In older children and adults, invasive pneumonia remained the most prevalent manifestation. The incidence of invasive pneumonia declined in the late post-implementation period in persons >65 years of age despite a significant increase in invasive pneumonia caused by non–PCV7 serotypes (Table 2).

**Table 2:** Incidence of invasive pneumococcal disease manifestations before and after implementation of a PCV7 vaccination program, the Netherlands, June 2004–May 2010*

Age group, y, manifestation	Incidence (%) by infecting serotype and vaccination period†							
	All serotypes			PCV7 serotypes			Non–PCV7 serotypes	
	Pre-implementation	Late post-implementation		Pre-implementation	Late post-implementation		Pre-implementation	Late post-implementation
<5								
Meningitis	6.80	3.88		4.80	0.43		2.00	3.45
Invasive pneumonia	4.40	1.72		3.00	0		1.40	1.72
Bacteremia other focus	3.60	1.29		2.80	0		0.80	1.29
Bacteremia without focus	3.80	1.29		2.40	0.22		1.40	1.08
5–64								
Meningitis	1.05	1.07		0.48	0.24		0.57	0.82
Invasive pneumonia	4.92	5.36		1.94	1.18		2.98	4.18
Bacteremia other focus	0.45	0.41		0.15	0.14		0.29	0.27
Bacteremia without focus	0.55	0.47		0.20	0.09		0.35	0.38
>65								
Meningitis	3.38	2.24		1.39	0.40		1.99	1.84
Invasive pneumonia	47.80	40.80		23.21	10.14		24.51	30.66
Bacteremia other focus	1.73	2.63		0.78	0.64		0.95	2.00
Bacteremia without focus	4.42	3.99		2.16	1.12		2.25	2.87

*Incidence is per 100,000 inhabitants. PCV7, 7-valent pneumococcal conjugate vaccine. †Vaccination periods: pre-implementation period, June 2004–May 2006; late post-implementation period, June 2008–May 2010.

Although the overall number of IPD cases declined from 1,216 in the pre-implementation period to 1,144 in the late post-implementation period, the number of IPD patients (all ages) with an immunocompromising condition increased from 216 to 255 (Table 3). This increase mainly occurred among persons >5 years of age, particularly among those >65 years of age. The number of PCV7-serotype IPD cases declined from 565 in the pre-implementation period to 268 in the late post-implementation period (all ages), and the number of patients with any comorbidity also showed a clear reduction. However, the number of immunocompromised persons with PCV7-serotype IPD declined only marginally (Table 3), indicating that persons with immunocompromising conditions may benefit less than others from herd immunity against PCV7-serotype IPD. This relatively marginal decline was seen for all PCV7 serotypes (data not shown). For non–PCV7 serotype IPD cases, there were similar increases in the number of infected immunocompromised patients and patients with any comorbidity. Moreover, at baseline a smaller proportion of immunocompromised (41%) than nonimmunocompromised (47%) persons had PCV7-serotype IPD (Table 4). Before and after introduction of PCV7, few children <5 years of age had a comorbid condition along with IPD (Technical Appendix Table).

**Table 3:** Characteristics for persons with invasive pneumococcal disease before and after implementation of a PCV7 vaccination program, the Netherlands, June 2004–May 2010*

Characteristic	No. (%) by infecting serotype and vaccination period†

All serotypes				PCV7 serotypes				Non–PCV7 serotypes		
Before (n = 1,216)	After (n = 1,144)	p value		Before(n = 565)	After (n = 268)	p value		Before (n = 650)	After (n = 876)	p value
Comorbidity											
Immunocompromising condition‡	216 (18)	255 (22)	**0.013**		88 (16)	73 (27)	**0.001**		128 (20)	182 (21)	NS
Any comobidity§	817 (67)	788 (69)	NS		376 (67)	190 (71)	NS		441 (68)	598 (68)	NS
Disease course/outcome											
ICU admission	258 (21)	243 (21)	NS		115 (20)	60 (22)	NS		143 (22)	183 (21)	NS
Length of hospital stay, median (IQR)	11.0 (7.0–18.0)	9.0 (5.0–16.0)	**<0.001**		11.0 (7.0–18.0)	9.0 (5.0–15.0)	**<0.001**		11.0 (7.0–19.0)	10.0 (5.0–16.0)	**<0.001**
Died	194 (16)	135 (12)	**0.003**		92 (16)	44 (16)	NS		102 (16)	91 (10)	**0.002**
Deaths/100,000 persons	2.4	1.6	**0.001**		1.1	0.5	**0.000**		1.3	1.1	NS

*Cases are number of patients included in a study covering ≈25% of the Dutch population. **Boldface,** significant difference (p<0.05) between pre- and post-implementation period as calculated by χ^2^ test (% of cases), Mann-Whitney U test (median days of hospitalization), or incidence rate ratio (mortality rate). PCV7, 7-valent pneumococcal conjugate vaccine; NS, not significant (p>0.05). †Data are no. (%) except as indicated in first column. Vaccination periods: before, pre-implementation period (June 2004–May 2006); after, late post-implementation period (June 2008–May 2010). ‡Immunocompromising condition: primary immunodeficiency, HIV/AIDS, lymphoma, leukemia, myeloma, solid organ or stem cell transplant, current immunosuppressive therapy for malignancy or autoimmune disease, asplenia/splenectomy, sickle cell disease, and renal insufficiency (dialysis required and nephrotic syndrome). §Any comorbidity: malignancies (within previous 5 y) not considered to be immunocompromising; chronic obstructive pulmonary disease; asthma; diabetes mellitus; myocardial infarction; coronary artery condition; stroke/transient ischemic attack; cardiomyopathy; heart failure; heart valve disease; presence of cerebral/abdominal/thoracic aneurysms; thyroid disease; liver disease; intravenous drug use; long-term alcohol abuse; cerebrospinal fluid leak; recent physical trauma/skull fracture; and, for children, premature birth (<37 weeks for children 0–1 y old and <32 weeks for children 0–4 y old).

**Table 4:** Proportion of vaccine-type and nonvaccine type invasive pneumococcal disease cases before and after implementation of a PCV7 vaccination program, the Netherlands, June 2004–May 2010*

Vaccination period, infecting serotype	No. (%) patients, by health status at time of infection				
	Otherwise healthy	Immunocompromising condition†	p value	Any comorbidity‡	p value
Pre-implementation period					
Total no. cases	399	216	NA	817	NA
PCV7 cases	189 (47)	88 (41)	NS	376 (46)	NS
Non–PCV7 cases	209 (52)	128 (59)	NS	441 (54)	NS
Post-implementation period					
Total no. cases	356	255	NA	788	NA
PCV7 cases	78 (22)	73 (29)	NS	190 (24)	NS
Non–PCV7 cases	278 (78)	182 (71)	**0.050**	598 (76)	NS

*Cases are number of patients included in a study covering ≈25% of the Dutch population. Pre-implementations period, June 2004–May 2006; post-implementation period, June 2008–May 2010. **Boldface,** significant difference (p<0.05, calculated by χ2 test) compared with otherwise healthy patients. PCV7, 7-valent pneumococcal conjugate vaccine; NA, not applicable; NS, not significant (p>0.05). †Immunocompromising condition: primary immunodeficiency, HIV/AIDS, lymphoma, leukemia, myeloma, solid organ or stem cell transplant, current immunosuppressive therapy for malignancy or autoimmune disease, asplenia/splenectomy, sickle cell disease, and renal insufficiency (dialysis required and nephrotic syndrome). ‡Any comorbidity: malignancies (within previous 5 y) not considered to be immunocompromising; chronic pulmonary disease (chronic obstructive pulmonary disease and asthma); diabetes mellitus; cardiovascular disease (myocardial infarction, coronary artery condition, stroke/transient ischemic attack, cardiomyopathy, heart failure, heart valve disease, and presence of cerebral/abdominal/thoracic aneurysms); thyroid disease; liver disease; intravenous drug use; long-term alcohol abuse; cerebrospinal fluid leak; recent physical trauma/skull fracture; and, for children, premature birth (<37 weeks for children 0–1 y old and <32 weeks for children 0–4 y old).

Despite the relative increase in immunocompromised patients with IPD, the overall death rate for IPD decreased significantly from 2.4 to 1.6 cases/100,000 persons. This decline in IPD-related deaths appears to be the result of 1) an overall decrease in the incidence of PCV7-serotype IPD and 2) a lower case-fatality rate among persons with non–PCV7 serotype IPD (Table 3). The lower death rate was seen in all age groups, but the decrease was significant only for patients >65 years of age. Moreover, a decrease in the case-fatality rate for non–PCV7 serotype cases was seen not only among otherwise healthy persons (decrease from 10% to 4%; p = 0.02), but also among immunocompromised persons (from 27% to 16%; p = 0.03) and/or persons with other comorbidities (from 19% to 14%; p = 0.03). Likewise, the median length of hospital stay for children >5 years of age and adults was significantly lower during the post-implementation period than in the pre-implementation period (Technical Appendix Table).

## Discussion

Our findings show that 4 years after introduction of PCV7 in the Netherlands, the overall annual incidence of IPD decreased by 60% (from 35.0 to 14.1 cases/100,000 persons) among children <2 years of age, the age group targeted for vaccination; the decrease was a result of virtually complete eradication of PCV7 serotypes. In children 2–4 years of age, a 48% reduction was seen in IPD cases overall. A significant decline of 13% was also observed in persons >65 years of age. No significant decline in overall IPD was seen in persons 5–64 years of age because the decline in PCV7-serotype IPD was offset by a similar increase in non–PCV7 serotype IPD. The proportion of immunocompromised patients within PCV7-serotype IPD also increased. Despite these findings, the length of hospital stay and case-fatality rates declined over the last years. Our findings indicate that use of PCV7 in the Netherlands resulted in a major decrease in PCV7-serotype IPD among all age groups.

Our results for children are in line with those in England and Wales (*4*). However, among persons 5–65 years of age, the effect of herd immunity was less pronounced in the Netherlands than in England and Wales (*4*), where PCV7 was introduced around the same time as in the Netherlands (summer 2006), or in the United States 4 years after the introduction of PCV7 in 2000 (*14*). This difference can be partly explained by the absence of a catch-up campaign for children <2 years of age in the Netherlands. Young children are a primary reservoir for carriage and transmission of pneumococci because of prolonged colonization episodes related to their immature immune systems. Vaccination of toddlers in addition to newborns has a major effect on the speed of onset of herd immunity in the population. Therefore, by continuing surveillance in the Netherlands, we will likely see more reduction of PCV7-serotype IPD in the years after 2010. A major issue will be the rise in non–PCV7 serotypes, which is estimated by Choi et al. (*19*) to be ≈90% in England and Wales. Despite this large increase in non–PCV7 serotype IPD, it is expected that this will not offset the decrease in PCV7-serotype IPD in infants and elderly persons.

The decline of IPD cases among persons with immunocompromising conditions was limited compared with the decline among nonimmunocompromised persons. This result may be biased because the number of PCV7-serotype IPD cases in this group was relatively small before introduction of PCV7. However, the case-fatality rate for non–PCV7 serotype IPD in the post-implementation period declined among otherwise healthy persons and among those with comorbid conditions, suggesting a less severe course of disease, even in patients with serious immunocompromising conditions. Thus, even if the incidence of IPD decreased less in immunocompromised persons than in the general population, persons with immunocompromising conditions still appear to benefit from the vaccination program because of a reduction in case-fatality rates.

The reduced case-fatality rate for non–PCV7 serotype IPD since the introduction of PCV7 can be partly explained by a large increase in serotype 1 IPD. This invasive serotype is associated with a low case-fatality rate (*12*,*15*,*20*), which remained low (6%–8%) in the Netherlands during the study period. Case-fatality rates for the other individual serotypes also did not change significantly after introduction of PCV7. In line with a lower case-fatality rate, we also found a reduced length of hospital stay for patients with PCV7-serotype IPD and those with non–PCV7 serotype IPD. However, in the Netherlands, there has been a tendency toward shorter hospital stays, which along with other factors (e.g., improved hospital efficiency) may affect the finding of a reduced length of hospital stay for patients with IPD (*21*). For example, in 2006 a new financial system was introduced in the Netherlands that encourages shortening of the length of hospital stay.

In children, the increase in non–PCV7 serotype disease was most pronounced among patients with meningitis. Although the numbers were too small to yield significant differences, these data indicate that surveillance should be continued and special attention should be paid to patient characteristics and the evolution of serotype circulation over time.

The incidence of IPD caused by nonvaccine– *S. pneumoniae* serotypes 1, 19A, 22F, and 23B increased significantly after introduction of PCV7 in the Netherlands. The increase in serotype 19A has been consistently reported worldwide, especially increased carriage among children (*22*,*23*) and increased cases of serotype 19A–associated invasive disease (*24*) and otitis media (*25*–*27*). The role of PCV7 in promoting serotype 19A carriage in vaccinated children compared with unvaccinated controls has been shown (*22*,*28*). In many countries, the increase in serotype 19A disease is associated with high levels of penicillin resistance (*24*). In the Netherlands, only 1.8% of pneumococcal strains are reported to be resistant (*29*). The increase in serotype 22F was also seen in the United States and in England and Wales (*3*,*4*). The occurrence of serotype 1 was also shown to fluctuate and decline in presence of PCV7 (*4*). We did not see an increase in IPD caused by serotypes 6C and 15B/C, although increases have been reported elsewhere (*3*,*4*). On May 1, 2011, the Dutch government switched from the 7-valent to the 10-valent pneumococcal conjugate vaccine, which includes serotypes 1, 5, and 7F in addition to those in PCV7 (*30*). The 13-valent pneumococcal conjugate vaccine, which has not been introduced in the Netherlands, adds protection against serotypes 3, 6A, and 19A.

Surveillance artifacts resulting from enhanced surveillance and increased awareness after the introduction of the vaccine should be considered when evaluating the effects of the PCV7 vaccination program (*4*). However, adjustments for these artifacts can introduce new biases leading to over- and underestimation of the true effects of the vaccine. We believe there are no indications for enhanced surveillance and increased awareness in our study. The laboratory-based surveillance system remained unchanged during the study period, 2004–2010. Unlike the situation in England and Wales (*4*), the number of pneumococcal isolates obtained from CSF samples in the Netherlands remained stable during the years before PCV7 was introduced (Technical Appendix Figure 1). Moreover, the incidences of IPD caused by a great majority of non–PCV7 serotypes remained stable during the entire study period; the exceptions were for IPD caused by serotypes 1, 19A, 22F, and 23B (Technical Appendix Figure 2). If enhanced surveillance had taken place, one would expect an increase in the reported number of IPD cases caused by any of these serotypes. Thus, we made no corrections for increased case ascertainment or awareness in this study.

Our study does have limitations. First, the study periods before and after implementation of the vaccine program were relatively short; this may have caused an overestimation or underestimation of our results. To account for a proper transition period, we did not include June 2006–May 2008 in our comparisons because no clear conclusions could be drawn from this period. Second, changes in IPD epidemiology could have been influenced by variations in the seasonal influenza and the influenza A(H1N1)pdm09 virus epidemics in 2009 (*31*,*32*). Last, no data were available on the national prevalence of comorbidities/diseases. Thus, we could not evaluate IPD incidence rate ratios for the 3 patient groups in our study: otherwise healthy persons, persons with any comorbidity, and persons with immunocompromising conditions.

The results of this study show that PCV7 use has reduced the number of IPD cases and deaths in children <2 years of age (the age group targeted for vaccination) and in persons >65 years of age. However, after introduction of PCV7, cases of IPD caused by non–PCV7 serotypes increased significantly among elderly persons, and the proportion of immunocompromised persons with IPD increased. Despite these increases, the overall IPD case-fatality rate among patients >65 years of age decreased, which seems to be a positive consequence of shifts in circulating serotypes after introduction of a pneumococcal conjugate vaccine for infants.

Technical Appendix Clinical characteristics of patients with invasive pneumococcal disease before and after introduction of 7-valent pneumococcal conjugate vaccine, nationwide collection of pneumococcal isolates, and incidence rate ratio of serotype-specific invasive pneumococcal disease among patients >65 years of age, the Netherlands.

## References

[1] Jansen AG, Rodenburg GD, de Greeff SC, Hak E, Veenhoven RH, Spanjaard L, Invasive pneumococcal disease in the Netherlands: syndromes, outcome and potential vaccine benefits. Vaccine. 2009;27:2394–401. 10.1016/j.vaccine.2009.01.12719428856

[2] Arguedas A, Soley C, Abdelnour A. Prevenar experience. Vaccine. 2011;29(Suppl 3):C26–34. 10.1016/j.vaccine.2011.06.10421896350

[3] Pilishvili T, Lexau C, Farley MM, Hadler J, Harrison LH, Bennett NM, Sustained reductions in invasive pneumococcal disease in the era of conjugate vaccine. J Infect Dis. 2010;201:32–41. 10.1086/64859319947881

[4] Miller E, Andrews NJ, Waight PA, Slack MP, George RC. Herd immunity and serotype replacement 4 years after seven-valent pneumococcal conjugate vaccination in England and Wales: an observational cohort study. Lancet Infect Dis. 2011;11:760–8. 10.1016/S1473-3099(11)70090-121621466

[5] Hammitt LL, Bruden DL, Butler JC, Baggett HC, Hurlburt DA, Reasonover A, Indirect effect of conjugate vaccine on adult carriage of *Streptococcus pneumoniae*: an explanation of trends in invasive pneumococcal disease. J Infect Dis. 2006;193:1487–94. 10.1086/50380516652275

[6] Pelton SI, Huot H, Finkelstein JA, Bishop CJ, Hsu KK, Kellenberg J, Emergence of 19A as virulent and multidrug resistant pneumococcus in Massachusetts following universal immunization of infants with pneumococcal conjugate vaccine. Pediatr Infect Dis J. 2007;26:468–72. 10.1097/INF.0b013e31803df9ca17529860

[7] Weinberger DM, Malley R, Lipsitch M. Serotype replacement in disease after pneumococcal vaccination. Lancet. 2011;378:1962–73. 10.1016/S0140-6736(10)62225-821492929PMC3256741

[8] Rozenbaum MH, Boersma C, Postma MJ, Hak E. Observed differences in invasive pneumococcal disease epidemiology after routine infant vaccination. Expert Rev Vaccines. 2011;10:187–99. 10.1586/erv.10.16321332268

[9] Weatherholtz R, Millar EV, Moulton LH, Reid R, Rudolph K, Santosham M, Invasive pneumococcal disease a decade after pneumococcal conjugate vaccine use in an American Indian population at high risk for disease. Clin Infect Dis. 2010;50:1238–46. 10.1086/65168020367225

[10] Lacapa R, Bliss SJ, Larzelere-Hinton F, Eagle KJ, McGinty DJ, Parkinson AJ, Changing epidemiology of invasive pneumococcal disease among White Mountain Apache persons in the era of the pneumococcal conjugate vaccine. Clin Infect Dis. 2008;47:476–84. 10.1086/59000118627249

[11] Rodenburg GD, de Greeff SC, Jansen AG, de Melker HE, Schouls LM, Hak E, Effects of pneumococcal conjugate vaccine 2 years after its introduction, the Netherlands. Emerg Infect Dis. 2010;16:816–23. 10.3201/eid1605.09122320409372PMC2953990

[12] Jansen AG, Rodenburg GD, van der Ende A, van Alphen L, Veenhoven RH, Spanjaard L, Invasive pneumococcal disease among adults: associations among serotypes, disease characteristics, and outcome. Clin Infect Dis. 2009;49:e23–9. 10.1086/60004519522653

[13] Brueggemann AB, Peto TE, Crook DW, Butler JC, Kristinsson KG, Spratt BG. Temporal and geographic stability of the serogroup-specific invasive disease potential of *Streptococcus pneumoniae* in children. J Infect Dis. 2004;190:1203–11. 10.1086/42382015346329

[14] Lexau CA, Lynfield R, Danila R, Pilishvili T, Facklam R, Farley MM, Changing epidemiology of invasive pneumococcal disease among older adults in the era of pediatric pneumococcal conjugate vaccine. JAMA. 2005;294:2043–51. 10.1001/jama.294.16.204316249418

[15] Weinberger DM, Harboe ZB, Sanders EA, Ndiritu M, Klugman KP, Ruckinger S, Association of serotype with risk of death due to pneumococcal pneumonia: a meta-analysis. Clin Infect Dis. 2010;51:692–9. 10.1086/65582820715907PMC2927802

[16] van Oosten M, de Greeff SC, Spanjaard L, Schouls LM. Introduction of pneumococcal conjugate vaccine into the Dutch national immunisation programme. Euro Surveill. 2006;11:E060608.2.1681911810.2807/esw.11.23.02968-en

[17] van Lier EA, Oomen PJ, Oostenbrug MWM, Zwakhals SLN, Drijfhout IH, de Hoogh PAAM, Vaccinatiegraad rijksvaccinatie programma Nederland; Verslagjaar 2009 [cited 2012 Jan 5]. http://www.rivm.nl/bibliotheek/rapporten/210021010.pdf

[18] de Greeff SC, Sanders EA, de Melker HE, van der Ende A, Vermeer PE, Schouls LM. Two pneumococcal vaccines: the 7-valent conjugate vaccine (Prevenar) for children up to the age of 5 years and the 23-valent polysaccharide vaccine (pneumo 23) for the elderly and specific groups at risk. Ned Tijdschr Geneeskd. 2007;151:1454–7.17633974

[19] Choi YH, Jit M, Gay N, Andrews N, Waight PA, Melegaro A, 7-valent pneumococcal conjugate vaccination in England and Wales: is it still beneficial despite high levels of serotype replacement? PLoS ONE. 2011;6:e26190. 10.1371/journal.pone.002619022022559PMC3193519

[20] Harboe ZB, Thomsen RW, Riis A, Valentiner-Branth P, Christensen JJ, Lambertsen L, Pneumococcal serotypes and mortality following invasive pneumococcal disease: a population-based cohort study. PLoS Med. 2009;6:e1000081. 10.1371/journal.pmed.100008119468297PMC2680036

[21] Borghans I, Heijink R, Kool T, Lagoe RJ, Westert GP. Benchmarking and reducing length of stay in Dutch hospitals. BMC Health Serv Res. 2008;8:220. 10.1186/1472-6963-8-22018950476PMC2582034

[22] Spijkerman J, van Gils EJ, Veenhoven RH, Hak E, Yzerman EP, van der Ende A, Carriage of *Streptococcus pneumoniae* 3 years after start of vaccination program, the Netherlands. Emerg Infect Dis. 2011;17:584–91.2147044510.3201/eid1704101115PMC3377405

[23] Dunais B, Bruno-Bazureault P, Carsenti-Dellamonica H, Touboul P, Pradier C. A decade-long surveillance of nasopharyngeal colonisation with *Streptococcus pneumoniae* among children attending day-care centres in south-eastern France: 1999–2008. Eur J Clin Microbiol Infect Dis. 2011;30:837–43. 10.1007/s10096-011-1154-921611871

[24] Reinert R, Jacobs MR, Kaplan SL. Pneumococcal disease caused by serotype 19A: review of the literature and implications for future vaccine development. Vaccine. 2010;28:4249–59. 10.1016/j.vaccine.2010.04.02020416266

[25] Stamboulidis K, Chatzaki D, Poulakou G, Ioannidou S, Lebessi E, Katsarolis I, The impact of the heptavalent pneumococcal conjugate vaccine on the epidemiology of acute otitis media complicated by otorrhea. Pediatr Infect Dis J. 2011;30:551–5. 10.1097/INF.0b013e31821038d921297521

[26] Hoberman A, Paradise JL, Shaikh N, Greenberg DP, Kearney DH, Colborn DK, Pneumococcal resistance and serotype 19A in Pittsburgh-area children with acute otitis media before and after introduction of 7-valent pneumococcal polysaccharide vaccine. Clin Pediatr (Phila). 2011;50:114–20. 10.1177/000992281038425921098526

[27] Fenoll A, Aguilar L, Vicioso MD, Gimenez MJ, Robledo O, Granizo JJ. Increase in serotype 19A prevalence and amoxicillin non-susceptibility among paediatric *Streptococcus pneumoniae* isolates from middle ear fluid in a passive laboratory-based surveillance in Spain, 1997–2009. BMC Infect Dis. 2011;11:239. 10.1186/1471-2334-11-23921910891PMC3180674

[28] van Gils EJ, Veenhoven RH, Hak E, Rodenburg GD, Keijzers WC, Bogaert D, Pneumococcal conjugate vaccination and nasopharyngeal acquisition of pneumococcal serotype 19A strains. JAMA. 2010;304:1099–106. 10.1001/jama.2010.129020823436

[29] SWAB. the Dutch Foundation of the Working Party on Antibiotic Policy. NethMap 2011: consumption of antimicrobial agents and antimicrobial resistance among medically important bacteria in the Netherlands [cited 2012 Jan 5]. http://www.swab.nl/swab/cms3.nsf/uploads/35ACD3A546C31716C12578BF002EDC4F/$FILE/NethMap2011.pdf

[30] National Institute for Public Health and the Environment. Invoering pneumokokkenvaccin Synflorix. Bilthoven (the Netherlands): the Institute; 2011.

[31] Martin-Loeches I, Sanchez-Corral A, Diaz E, Granada RM, Zaragoza R, Villavicencio C, Community-acquired respiratory coinfection in critically ill patients with pandemic 2009 influenza A(H1N1) virus. Chest. 2011;139:555–62. 10.1378/chest.10-139620930007

[32] Wielders CC, van Lier EA, van ’t Klooster TM, van Gageldonk-Lafeber AB, van den Wijngaard CC, Haagsma JA, The burden of 2009 pandemic influenza A(H1N1) in the Netherlands. Eur J Public Health. 2012;22:150–7. 10.1093/eurpub/ckq18721183472

# Research article: Invasive Pneumococcal Disease and 7-Valent Pneumococcal Conjugate Vaccine, the Netherlands

**Authors:** Anna M.M. van Deursen, Suzan P. van Mens, Elisabeth A.M. Sanders, Bart J.M. Vlaminckx, Hester E. de Melker, Leo M. Schouls, Sabine C. de Greeff, Arie van der Ende, on behalf of the Invasive Pneumococcal Disease Sentinel Surveillance Laboratory Group

## Medscape CME Quiz

## Earning CME Credit

To obtain credit, you should first read the journal article. After reading the article, you should be able to answer the following, related, multiple-choice questions. To complete the questions (with a minimum 70% passing score) and earn continuing medical education (CME) credit, please go to www.medscape.org/journal/eid. Credit cannot be obtained for tests completed on paper, although you may use the worksheet below to keep a record of your answers. You must be a registered user on Medscape.org. If you are not registered on Medscape.org, please click on the New Users: Free Registration link on the left hand side of the website to register. Only one answer is correct for each question. Once you successfully answer all post-test questions you will be able to view and/or print your certificate. For questions regarding the content of this activity, contact the accredited provider, CME@medscape.net. For technical assistance, contact CME@webmd.net. American Medical Association’s Physician’s Recognition Award (AMA PRA) credits are accepted in the US as evidence of participation in CME activities. For further information on this award, please refer to http://www.ama-assn.org/ama/pub/category/2922.html. The AMA has determined that physicians not licensed in the US who participate in this CME activity are eligible for *AMA PRA Category 1 Credits*™. Through agreements that the AMA has made with agencies in some countries, AMA PRA credit may be acceptable as evidence of participation in CME activities. If you are not licensed in the US, please complete the questions online, print the certificate and present it to your national medical association for review.

## Article Title:  Invasive Pneumococcal Disease and 7-Valent Pneumococcal Conjugate Vaccine, the Netherlands

## CME Questions

**1. You are seeing a 2-month-old infant whose parents have multiple questions regarding the effects of the 7-valent pneumococcal conjugate vaccine (PCV7). What does previous research demonstrate regarding the efficacy of PCV7?**

A. PCV7 primarily reduces the risk of otitis media among children

B. PCV7 has no effect on the rate of invasive pneumococcal disease (IPD) among adults

C. PCV7 has no effect on the rate of IPD among young children

D. The rate of non-PCV7-serotype IPD increased after introduction of PCV7

**2. What else should you consider as you discuss PCV7 with these parents?**

A. IPD incidence has declined to a greater degree in Europe vs the United States

B. Catch-up vaccination programs for children under 5 have sped up eradication of vaccine serotypes

C. The greatest indirect benefit of PCV7 is found among older adults with comorbid conditions

D. PCV7 contains serotype 1, which is the most common serotype implicated in IPD

**3. As you continue your conversation regarding PCV7, which age groups from the current study can you tell these parents experienced the greatest decline in the incidence of IPD?**

A. 2 to 4 years of age and 5 to 12 years of age

B. 0 to 2 years of age and middle-aged adults

C. 0 to 2 years of age and 65 years or older

D. 21 to 35 years of age and 50 to 64 years of age

**4. What can you tell these parents regarding characteristics of IPD in the current study?**

A. IPD incidence due to non–PCV7-serotypes increased among young children but fell among adults

B. Pneumonia was the most common infection related to IPD among children less than 5 years of age

C. PCV-7 appeared particularly effective in reducing IPD among immunocompromised individuals

D. The overall mortality rate associated with IPD declined during the study period

## Activity Evaluation

**Table:** 

**1. The activity supported the learning objectives.**				
Strongly Disagree				Strongly Agree
1	2	3	4	5
**2. The material was organized clearly for learning to occur.**				
Strongly Disagree				Strongly Agree
1	2	3	4	5
**3. The content learned from this activity will impact my practice.**				
Strongly Disagree				Strongly Agree
1	2	3	4	5
**4. The activity was presented objectively and free of commercial bias.**				
Strongly Disagree				Strongly Agree
1	2	3	4	5
